# DODGE: automated point source bacterial outbreak detection using cumulative long term genomic surveillance

**DOI:** 10.1093/bioinformatics/btae427

**Published:** 2024-07-02

**Authors:** Michael Payne, Dalong Hu, Qinning Wang, Geraldine Sullivan, Rikki M Graham, Irani U Rathnayake, Amy V Jennison, Vitali Sintchenko, Ruiting Lan

**Affiliations:** School of Biotechnology and Biomolecular Sciences, University of New South Wales, Sydney, NSW 2052, Australia; School of Biotechnology and Biomolecular Sciences, University of New South Wales, Sydney, NSW 2052, Australia; Centre for Infectious Diseases and Microbiology—Public Health, Institute of Clinical Pathology and Medical Research—NSW Health Pathology, Westmead Hospital, Sydney, NSW 2145, Australia; Centre for Infectious Diseases and Microbiology—Public Health, Institute of Clinical Pathology and Medical Research—NSW Health Pathology, Westmead Hospital, Sydney, NSW 2145, Australia; Public Health Microbiology, Queensland Health Forensic and Scientific Services, Coopers Plains, Brisbane, QLD 4108, Australia; Public Health Microbiology, Queensland Health Forensic and Scientific Services, Coopers Plains, Brisbane, QLD 4108, Australia; Public Health Microbiology, Queensland Health Forensic and Scientific Services, Coopers Plains, Brisbane, QLD 4108, Australia; Centre for Infectious Diseases and Microbiology—Public Health, Institute of Clinical Pathology and Medical Research—NSW Health Pathology, Westmead Hospital, Sydney, NSW 2145, Australia; Sydney Institute for Infectious Diseases, Sydney Medical School, University of Sydney, Sydney, NSW 2006, Australia; School of Biotechnology and Biomolecular Sciences, University of New South Wales, Sydney, NSW 2052, Australia

## Abstract

**Summary:**

The reliable and timely recognition of outbreaks is a key component of public health surveillance for foodborne diseases. Whole genome sequencing (WGS) offers high resolution typing of foodborne bacterial pathogens and facilitates the accurate detection of outbreaks. This detection relies on grouping WGS data into clusters at an appropriate genetic threshold. However, methods and tools for selecting and adjusting such thresholds according to the required resolution of surveillance and epidemiological context are lacking. Here we present DODGE (Dynamic Outbreak Detection for Genomic Epidemiology), an algorithm to dynamically select and compare these genetic thresholds. DODGE can analyse expanding datasets over time and clusters that are predicted to correspond to outbreaks (or “investigation clusters”) can be named with established genomic nomenclature systems to facilitate integrated analysis across jurisdictions. DODGE was tested in two real-world *Salmonella* genomic surveillance datasets of different duration, 2 months from Australia and 9 years from the United Kingdom. In both cases only a minority of isolates were identified as investigation clusters. Two known outbreaks in the United Kingdom dataset were detected by DODGE and were recognized at an earlier timepoint than the outbreaks were reported. These findings demonstrated the potential of the DODGE approach to improve the effectiveness and timeliness of genomic surveillance for foodborne diseases and the effectiveness of the algorithm developed.

**Availability and implementation:**

DODGE is freely available at https://github.com/LanLab/dodge and can easily be installed using Conda.

## 1 Introduction

Foodborne pathogens are a major cause of morbidity globally with 550 million infections reported in 2010 (Kirk *et al.* 2015). *Salmonella enterica* is a common cause of these infections with 78 million cases per year with the two most common serovars being *S.* Typhimurium (STM) and *S.* Enteritidis (SEN) (Hendriksen *et al.* 2011, Kirk *et al.* 2015). Once they reach the human population from agricultural and environmental reservoirs the control of these pathogens depends on the identification and elimination of outbreaks. Outbreaks are mostly caused by single strains that contaminate food and lead to many cases of disease over a short time span. The identification of an outbreak has therefore relied on identifying strains that share the same genetic or phenotypic makeup and have occurred over a short temporal window (Sabat *et al.* 2013).

Whole genome sequencing (WGS) has offered new capacity to identify related clinical and food/source isolates at high resolution. Previous studies have demonstrated that isolates within an outbreak examined using WGS are often not genetically identical but are very closely related (Octavia *et al.* 2015). Therefore, there is a need to group isolates together using a genetic distance threshold. Tools exist to group related isolates together at a variety of thresholds but provide no means to select a threshold for outbreak detection (Dallman *et al.* 2018, Lees *et al.* 2019, Zhou *et al.* 2021). Static thresholds have been applied in multiple studies (Octavia *et al.* 2015, Bekal *et al.* 2016, Phillips *et al.* 2016, Gymoese *et al.* 2017, Tolar *et al.* 2019, Paranthaman *et al.* 2021, Stevens *et al.* 2022). However, a single static genetic threshold without including other metadata is unlikely to be universally applicable due to differences in genetic diversity across bacterial populations and differences in the transmission pathways within an outbreak (Octavia *et al.* 2015, Phillips *et al.* 2016, Payne *et al.* 2019). We previously demonstrated the utility of a variable genetic threshold that depended on the local diversity of isolates over time and provided optimal sensitivity and specificity for outbreak detection (Payne *et al.* 2019). Therefore, there is a need for a method and software tool that can identify potential outbreak clusters using thresholds determined dynamically based on the population and evolutionary dynamics of the pathogen.

Public health genomic surveillance has become routine in many countries, therefore any software solution must also be capable of identifying and tracking outbreaks over time in continuously expanding datasets. A recent study provided a system for naming and tracking of genomic clusters over time but did not provide a means to identify them (Mixão *et al.* 2023). Another promising approach employs temporal metadata and evolutionary modelling to select optimal genetic clusters but did not provide a system for continuous surveillance (Duval *et al.* 2023).

In this study we present DODGE (Dynamic Outbreak Detection for Genomic Epidemiology), a method and tool to identify outbreaks with dynamic genetic thresholds selected using temporal thresholds that can accommodate expanding datasets from ongoing surveillance (software available from https://github.com/LanLab/dodge). This method utilizes retrospective genomic surveillance data to define a background dataset which is then used to identify distinct, new clusters that subsequently appear. The method was tested on one Australian dataset, all STM isolates from a 2-month period from two states, and three United Kingdom datasets of STM, SEN, and *Shigella flexneri* isolates over 8 or 9 years.

## 2 Materials and methods

### 2.1 DODGE inputs

DODGE is primarily designed for use with core genome multilocus sequencing typing (cgMLST) allele profiles and can accept this data directly downloaded from MGTdb (https://www.mgtdb.unsw.edu.au/) or Enterobase (https://enterobase.warwick.ac.uk/) (Payne *et al.* 2020, Zhou *et al.* 2020, Kaur *et al.* 2022). Temporal and nomenclature data (Multilevel genome typing (MGT), sequence types (STs) or hierCC clusters) are extracted from metadata files that can be obtained from the corresponding databases. To facilitate *ad*-*hoc* analyses using DODGE, single nucleotide polymorphism (SNP) based inputs can also be used. Inputs using SNP analysis are vcf files and masked genomes produced by the program snippy (Seemann 2015, https://github.com/tseemann/snippy). To maximize the specificity of DODGE a background dataset is required that should ideally include a set of isolates immediately prior to the period to be analysed for potential outbreaks.

### 2.2 DODGE pipeline

**Figure 1.**
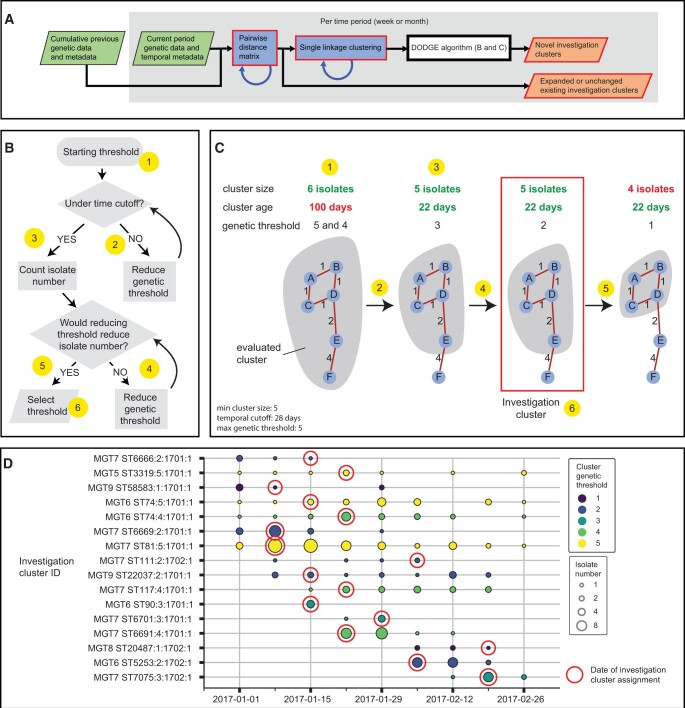
The DODGE pipeline, algorithm, and Australian dataset investigation clusters. (a) High level schematic of the DODGE pipeline including the DODGE algorithm. Genetic data in the form of allele profiles (Enterobase or MGTdb) or SNPs (output by snippy) for isolates from a given time period (a week or month) are combined with previous time periods to generate a genetic distance matrix. Distances between isolate pairs that are not in an optional input distance matrix (blue circular arrow) are calculated and added. The distance matrix is used to identify new isolates for existing investigation clusters. Single linkage clustering is performed on isolates not in investigation clusters. Single linkage clusters are then used to identify novel investigation clusters using the DODGE algorithm detailed in b and c. Green boxes on the left are input files, red outlined boxes on the right are output files, blue circular arrows represent outputs from one time period used as inputs in the next. (b) Flowchart describing six stages of the DODGE algorithm. (c). Example investigation cluster detection with the same six stages marked. Blue circles with letters represent isolates, and numbers beside the red lines that connect the circles are genetic distances. At each genetic threshold isolates within the grey shaded area are the cluster being evaluated. (d) Investigation clusters identified from the Australian dataset over time. *X* axis is date of collection by week. *Y* axis is investigation cluster with MGT ST based ID. The area of circles is proportional to number of isolates in that investigation cluster in that week. Colour represents the genetic threshold selected for the investigation cluster when it is first called as an investigation cluster. Red outline surrounding a circle indicates the week in which the cluster was identified as an investigation cluster by the DODGE algorithm.

To detect outbreaks from continuous pathogen surveillance, genomic analysis must accommodate new isolates accumulated on a regular basis. For this reason, the DODGE pipeline divides the input dataset into a series of user defined time periods (e.g. 1 week or 1 month) and detects potential outbreaks (termed investigation clusters) once for each time period (stages of the DODGE pipeline described in Fig. 1a). For example, if a dataset contains 2 months of data and the time window was set to weekly then the DODGE pipeline would be run once on the background dataset to establish the genetic background then run once for each week within the 2 months. Each weekly detection run would use the background and previous time periods’ outputs as inputs into the DODGE algorithm to identify new investigation clusters and describe any existing investigation clusters that have expanded from one week to the next. Importantly investigation cluster names assigned by the DODGE algorithm are inherited across time periods to allow ongoing surveillance and tracking of the clusters. The DODGE pipeline can also be run with a single static genetic threshold instead of the DODGE algorithm if desired.

The stages of the DODGE pipeline that are carried out per time period are as follows. A pairwise distance matrix is generated from either allelic profiles or SNPs. Isolates that match to existing investigation clusters at that cluster’s genetic threshold are added to that cluster. Single linkage clustering is applied to all isolates not in existing investigation clusters (including previous time periods and background data). These single linkage clusters and metadata are used as input into the DODGE algorithm.

### 2.3 DODGE algorithm

To identify genetic clusters of isolates that are likely to correspond to point source outbreaks, DODGE uses a temporal threshold to select the genetic threshold for each investigation cluster independently. The stages of the DODGE algorithm are as follows (Fig. 1b and c). Select all genetic clusters at the maximum single linkage distance set by user (default of five alleles or SNPs). For each cluster, keep if it is above the user defined minimum size (default of five isolates or cases) and record its timespan. If the timespan is greater than the user defined temporal threshold (default of 28 days), reduce the genetic threshold by one allele or SNP and check temporal threshold again. This is repeated until the timespan of the cluster is below the temporal threshold. This cluster is then assigned as an investigation cluster which denotes a potential outbreak that may warrant public health investigation. The minimum genetic threshold that retains the initial investigation cluster size is selected. Additionally, the temporal threshold used for initial investigation cluster identification is not applied in subsequent time periods, allowing long lived investigation clusters to be reported.

Each investigation cluster is named using four characteristics: the genomic identity of its constituent isolates at the time of detection (e.g. MGT ST or hierCC cluster), the genetic threshold used to identify it, the year and month it was assigned as an investigation cluster, and a unique integer identifier to differentiate any otherwise indistinguishable names. The full cluster name is therefore “genomic identity: genetic threshold: YearMonth: unique integer identifier.” For *ad-hoc* SNP analyses the genomic identity is not assigned.

For the temporal window to be effective in selecting genetic thresholds the background dataset is essential. The inclusion of this dataset allows DODGE to identify genetic clusters that span from the background time period into the detection time period and exclude them as new outbreaks.

Any genetic cluster that originated in the background will trigger DODGE to reduce the genetic threshold until no background isolates are included ensuring that newly emerged clusters are detected as opposed to long lived endemic clones.

### 2.4 DODGE case study datasets and algorithm settings

All analyses used DODGE version 1.0.0. The Australian dataset includes genomic data for all STM isolates collected and sequenced at New South Wales (NSW) and Queensland (QLD) public health laboratories in January and February 2017. All isolates in the STM MGTdb from Australia before 2017 were used as the background dataset. DODGE was run on this dataset with five isolate minimum cluster size and 28-day temporal threshold (i.e. five cases in a 28-day window as signal of an outbreak) and an initial genetic threshold of five.

For the United Kingdom datasets all STM and SEN isolates from the United Kingdom from 2014 to 2022 that had year and month metadata were extracted from the STM MGTdb database. DODGE was run using a minimum cluster size of five isolates, a maximum genetic distance of five alleles and a 2-month temporal window. All isolates from 2014 were used as background data in each dataset.

The *S. flexneri* dataset included all United Kingdom isolates in Enterobase that included year and month metadata from 2015 to 2022. DODGE settings were identical to the SEN dataset except for adding the “--enterobase_data” flag.

Cohen’s Kappa (Cohen 1960) was used to measure the agreement of DODGE assignments between SNP and allele input data. The metric ranges from less than 0 meaning no better agreement than expected by chance and 0.8–1 meaning almost perfect agreement.

## 3 Results

### 3.1 Application to 2 months of Australian STM surveillance data

A total of 517 STM genomes from NSW and QLD sequenced in January and February 2017 were used to identify investigation clusters using DODGE (data available at https://github.com/LanLab/dodge/tree/main/examples/). Existing publicly available Australian isolates collected prior to 2017 were used as background data and included 1030 isolates over 26 years (Supplementary Table S1). Sixteen investigation clusters including 245 isolates (47.3%) were identified from the 2 months of surveillance (Table 1, Fig. 1d, Supplementary Fig. S1, Supplementary Table S2). No information was available to confirm the number or composition of epidemiologically investigated outbreaks in this dataset.

**Table 1.** Characteristics of the application datasets and DODGE investigation clusters identified in them.

Serovar	Genomes	Investigation cluster characteristics				% of investigation cluster isolates relative to identification date		
		Number of clusters	Total isolates (%)	Average timespan (months)	Average size	Before	On date	After
Australian *S.*Typhimurium	517	16	245 (47.3%)	29.1	15.3	15.5	34.7	49.8
United Kingdom *S.*Typhimurium	11 841	111	1982 (16.5%)	8.6	19.1	6.5	40.3	53.2
United Kingdom *S.*Enteritidis	17 055	229	4236 (24.8%)	11.7	19.2	6.4	33.6	60.0
United Kingdom *S. flexneri*	3766	11	296 (7.8%)	9.7	26.9	3.7	20.9	75.3

To evaluate the agreement between allele and SNP based outputs of DODGE, the Australian dataset was also run using SNP inputs. Investigation clusters from the two methodologies showed good agreement with a Cohen’s kappa score of 0.743 (Supplementary Results).

### 3.2 Application to 9 years of United Kingdom STM and SEN genomic surveillance data

Genomic surveillance in the United Kingdom represents the largest complete dataset that had month and year metadata (Supplementary Tables S3 and S5). Publicly available STM and SEN data within their respective MGT databases between 2014 and 2022 were evaluated using DODGE (STM 13 091 genomes, SEN 18 972 genomes) (Payne *et al.* 2020, Luo *et al.* 2021, Kaur *et al.* 2022). Isolates from 2014 were used as background data and the remaining 8 years of isolates were used to detect 111 and 229 investigation clusters for STM and SEN, respectively (Table 1, Supplementary Figs S1 and S2, Supplementary Tables S4 and S6).

Most investigation clusters could not be verified as no information on the composition of epidemiologically confirmed outbreaks was available. However, two epidemiologically confirmed STM outbreaks could be matched to publicly available representative isolates within the United Kingdom dataset. The first was reported by public health authorities in April 2020 and caused 104 confirmed cases in the United Kingdom (European Centre for Disease Prevention and Control 2020). A representative from this cluster fell within the MGT9 ST22592:1:2002:1 investigation cluster which contained 90 isolates and was assigned as an investigation cluster in February 2020.

The second outbreak was reported by public health authorities in February 2022 and consisted of two distinct clusters which caused 102 and 7 epidemiologically linked cases in the United Kingdom, respectively (Larkin *et al.* 2022). Two representatives for Cluster 1 fell within investigation cluster MGT8 ST28184:1:2201:1, which contained 102 isolates and was assigned as an investigation cluster in January 2022. Two representatives for Cluster 2 fell within investigation cluster MGT9 ST30910:3:2203:1, which contained seven isolates and was assigned as an investigation cluster March 2022.

### 3.3 Application to 8 years of *S.flexneri* United Kingdom genomic surveillance data

To examine the performance of DODGE in a different species, we analysed 4114 *S. flexneri* genomes from the United Kingdom and applied the hierCC nomenclature implemented in Enterobase (Zhou *et al.* 2020, 2021). The background dataset included 348 isolates from 2015. Isolates from 2016 to 2022 (*N* = 3766) were analysed for investigation clusters. DODGE called 11 investigation clusters (Table 1, Supplementary Fig. S3, Supplementary Tables S7 and S8) with HC2 83474:4:1902:1 being the largest (174 genomes), persisting from February 2019 onwards.

### 3.4 Comparison of DODGE to a static threshold

The performance of a static threshold of five allele differences was also evaluated. This resulted in a 1.2–3.2 fold reduction of the number of investigation clusters called while increasing the timespan of investigation clusters by 1.1–2 fold depending on the dataset (Supplementary Results). Therefore, a static threshold may lack the sensitivity to detect new outbreak clusters caused by endemic homogenous clones.

## 4 Discussion

The identification of point source outbreaks using automated methods often rely on epidemiological data such as time, location, and strain phenotype/genotype without considering detailed genetic relationships between isolates (Salmon *et al.* 2016, Latash *et al.* 2020, Zhang *et al.* 2021). The increased uptake of WGS for routine prospective public health surveillance of different bacterial pathogens has the potential to provide this genetic context. However, the identification and reporting of emerging outbreaks from large datasets requires significant time and expertise.

DODGE is designed to identify a potential outbreak cluster by dynamically selecting the genetic threshold appropriate for the given investigation cluster using large, long term ongoing genomic surveillance datasets. This is achieved by identifying a genetic threshold for a given cluster that is stringent enough to exclude all isolates that occur more than a certain time in the past. In this way when sufficient background data is available, DODGE can adapt to the diversity of the bacterial population to provide accurate outbreak cluster detection.

The United Kingdom STM background dataset consisted of comprehensive genomic surveillance of the year immediately prior to the investigation period while the Australian STM background dataset was based on sporadic published data from any time before 2017. This difference likely explained the larger proportion of STM isolates included in investigation clusters in the Australian dataset (47.3% in Australian dataset, 16.5% in United Kingdom dataset). Persistent, low diversity clusters may not have been sampled in the Australian background STM population and these would appear as new investigation clusters in the 2 months data to which DODGE was applied. Even when high quality background data is available, if a persistent low diversity cluster was not sampled in the background (either because it was not sampled or did not yet exist), it will not be distinguishable from a new point source outbreak. Such persistent investigation clusters could also lose sensitivity over time if new isolates diverge from all existing cluster isolates by more than the cluster genetic threshold. However, once an investigation cluster has persisted for a long period it is likely that detailed epidemiological analysis would have been performed to track its original source.

Other key features of DODGE are the ability to analyse data in on-going surveillance while maintaining cluster identity through existing bacterial genomic nomenclature systems (such as MGT and hierCC). These nomenclature systems have been applied to all public global data for STM allowing investigation clusters to be placed in broader genomic context while also facilitating simple communication of outbreak types. The snapperDB genomic nomenclature system is based on SNP distances and could be integrated with DODGE using precomputed distance matrices. However, there is no publicly available database for this system and thus its application was not implemented or tested in this study.

Two investigation clusters from the United Kingdom STM dataset were matched with previously described outbreaks (European Centre for Disease Prevention and Control 2020, Larkin *et al.* 2022). In both cases, representative isolates from the outbreaks were found within investigation clusters predicted by DODGE. These investigation clusters matched the size and timeframe reported for the outbreaks. Importantly, if we allow 1 month for genome sequencing to be performed in both cases, investigation clusters were identified at or prior to the date the cluster was originally reported (the same month for MGT8 ST28184:1:2201:1, and one month earlier for MGT9 ST22592:1:2002:1). While any genomic method could also theoretically identify outbreaks in this timeframe, DODGE is automated and objective, removing the need for possibly subjective manual examination of the clustering of isolates. In all datasets, a significant proportion of isolates in investigation clusters were sampled after their respective investigation clusters were first detected (between 49.8% and 75.3%). If these clusters were investigated in a timely manner and preventative measures were implemented, the public health and societal burden of such clusters could be substantially reduced.

*S. flexneri* in the United Kingdom is associated with either travel or men who have sex with men (MSM) (Bardsley *et al.* 2020). A recent study described a multidrug resistant (MDR) clade associated with MSM that emerged in the United Kingdom in 2019 and persisted throughout the study (named CC245-1580) (Dallman *et al.* 2021). This clade was accurately detected by DODGE (86 of 95 genomes in the study were assigned to investigation cluster HC2 83474:4:1902:1) one month after its appearance. This demonstrated the usefulness of DODGE in detecting the emergence of persistent clusters in a different species and, in the case of this outbreak, with a different mode of transmission. This example also demonstrated the applicability of the hierCC nomenclature (available in Enterobase) to outbreak naming with DODGE.

DODGE provides an automated means to identify, name and track outbreak clusters using dynamic thresholds from prospective genomic surveillance datasets and can be incorporated within laboratory surveillance and analysis workflows. DODGE is publicly available (https://github.com/LanLab/dodge) and could be used to accelerate the identification and control of point source outbreaks in any bacterial species where appropriate quality surveillance data is available.

## Supplementary Material

btae427_Supplementary_Data

## Data Availability

All input data is available from the MGTdb website (mgtdb.unsw.edu.au) and the DODGE github page (https://github.com/LanLab/dodge).
