# Supplementary material for: DODGE: automated point source bacterial outbreak detection using cumulative long term genomic surveillance
Source: Bioinformatics. 2024 Jul 2;40(7):btae427. doi: 10.1093/bioinformatics/btae427 (PMC11244691; doi:10.1093/bioinformatics/btae427)
Supplement: btae427_Supplementary_Data [file btae427_supplementary_data.zip › Supplementary results.docx]

**Supplementary methods**

**Genomic nomenclature used in investigation cluster identifiers**

For cgMLST data obtained from the MGTdb website each investigation cluster was assigned an MGT ST. For data obtained from Enterobase a hierCC cluster name was assigned (Zhou *et al.* 2021). The name was selected at the highest resolution level where greater than 70% of isolates in the cluster shared the same ST (for MGT) or cluster (for hierCC). For example, in a cluster of 20 isolates, 20 (100%) had the same MGT6 ST, 17 (85%) had the same MGT7 ST and 12 (60%) had the same MGT8 ST, the MGT7 ST was then chosen as the investigation cluster name. The same process was used for hierCC progressing from larger to smaller threshold hierCC clusters. Because ad-hoc SNP based analyses have no standardised nomenclature, numerical investigation cluster names were assigned per analysis.

**Supplementary results**

**Relative performance of SNP and cgMLST data in the Australian dataset**

To evaluate the relative performance of SNPs and cgMLST alleles (from MGTdb) Australian dataset was also analysed using SNP based analyses. SNPs were identified from raw Illumina reads for all isolates using snippy version 4.6.0 with default settings. In the 2 month dataset SNPs have slightly higher resolution than MGT alleles due to the cgMLST scheme of MGT9 covering only 82% of the LT2 reference genome. Indeed 82.2% of SNPs were called in genomic regions included in the cgMLST scheme. Agreement of genetic variant assignment (alleles vs SNPs called in the corresponding locus) between cgMLST and SNPs was 94.7% with only 2.0% of variants unique to MGT and 3.3% of variants unique to snippy. Using SNP data, 12 investigation clusters with 213 isolates were identified. All 12 of these outbreaks were also identified when DODGE was run using MGT allele profiles. MGT identified 16 clusters in total. This 4 cluster discrepancy is composed three clusters that were not detected using SNPs (MGT5 ST3319:5:1701:1, MGT6 ST74:5:1701:1, MGT6 ST90:3:1701:1) as well as two MGT based clusters that have merged to become one cluster in the SNP analysis (MGT8 ST20487:1:1702:1, MGT9 ST22037:2:1701:1). Of the clusters that were detected by both methods (including the 2 that merged) 4 were identical, 2 were larger in the MGT analysis, 3 were larger in the SNP analysis and 3 had unique isolates from both methods. Overall, 39 isolates were uniquely identified in MGT, 12 isolates were uniquely identified by SNPs and 199 isolates were identified by both. The overall agreement in investigation cluster assignment as evaluated by Cohen’s kappa was 0.743 and overall, 13 of 16 MGT clusters were identified by SNP analysis and 12 of 12 SNP clusters were identified in MGT analysis.

**Performance of static thresholds in comparison to DODGE algorithm**

To evaluate the benefit that DODGE provides over a simple static genetic threshold, all four datasets were analysed with a static threshold of 5 allele difference both with and without the temporal window applied. If the temporal window was applied (all isolates in a cluster must initially fall within the window) fewer investigation clusters containing fewer total isolates are called relative to DODGE, however average cluster timespans are longer (Supplementary results Table 1). When the temporal window was not applied, far more isolates were included in more investigation clusters and these clusters had an even longer average timespan. Given that point source outbreaks are often short lived having a shorter average investigation cluster timespan including more isolates is the desired outcome.

The magnitude of the differences between DODGE and static threshold approaches change between the datasets. This is most likely caused by two factors. The first being how complete and temporally close the background dataset is to the dataset being examined. A background dataset that is more temporally and genetically similar to the detection dataset will cause DODGE to use lower and more precise thresholds. Outbreaks within long lived clones that are present in the background will only be described with the DODGE algorithm, not a static threshold, increasing the number of investigation clusters called by DODGE relative to a static threshold. The second is overall homogeneity of the population of each dataset. In a more diverse dataset, a larger threshold (such as 5 allele differences) may be sufficient to separate outbreak from background data. In that situation the advantage of DODGE is reduced as outbreaks could be identified with a static threshold of 5 without needing further refinement with the DODGE algorithm. A rough measure of the homogeneity of a dataset is the proportion of isolates that fall in investigation clusters using a static genetic threshold with no temporal threshold (I.e. the proportion of the dataset in simple 5 threshold genetic clusters). The UK SEN surveillance dataset has 79% of its isolates in these clusters while for the UK *S. flexneri* surveillance dataset the value is only 46% suggesting the SEN dataset is much more homogeneous. This homogeneity difference then leads to differences in the magnitude of the advantage DODGE has over a static threshold. For the UK SEN dataset DODGE included 2.7 times more isolates in 3.2 times more outbreaks than a static threshold of 5 alleles while these values were only 1.06 and 1.6 for *S. flexneri*.

Supplementary Results Table 1. Relative performance of DODGE and static thresholds.

| **Dataset** | **Total Dataset** | **Dodge** | | | **Static** | | | **Static + no temporal threshold** | | |
| --- | --- | --- | --- | --- | --- | --- | --- | --- | --- | --- |
|  |  | **Clusters** | **Isolates** | **Average timespan*** | **Clusters** | **Isolates** | **Average timespan*** | **Clusters** | **Isolates** | **Average timespan*** |
| Aus STM | 517 | 16 | 245 | 29.1 days | 13 | 222 | 31.4 days | 17 | 316 | 318 days |
| UK STM | 11841 | 111 | 1982 | 8.6 m | 76 | 1242 | 17.5 m | 308 | 5433 | 29.8 m |
| UK SEN | 17055 | 229 | 4236 | 11.7 m | 72 | 1543 | 16.4 m | 410 | 13495 | 34.1 m |
| UK SF | **3766** | **11** | **296** | **9.7 m** | 7 | 279 | 14.7 m | 69 | 1729 | 20.2 m |

* Average timespans are in months for UK datasets and in days for the Australian dataset.
