# Supplementary material for: DODGE: automated point source bacterial outbreak detection using cumulative long term genomic surveillance
Source: Bioinformatics. 2024 Jul 2;40(7):btae427. doi: 10.1093/bioinformatics/btae427 (PMC11244691; doi:10.1093/bioinformatics/btae427)
Supplement: btae427_Supplementary_Data [file btae427_supplementary_data.zip › Supplementary figures.pdf]

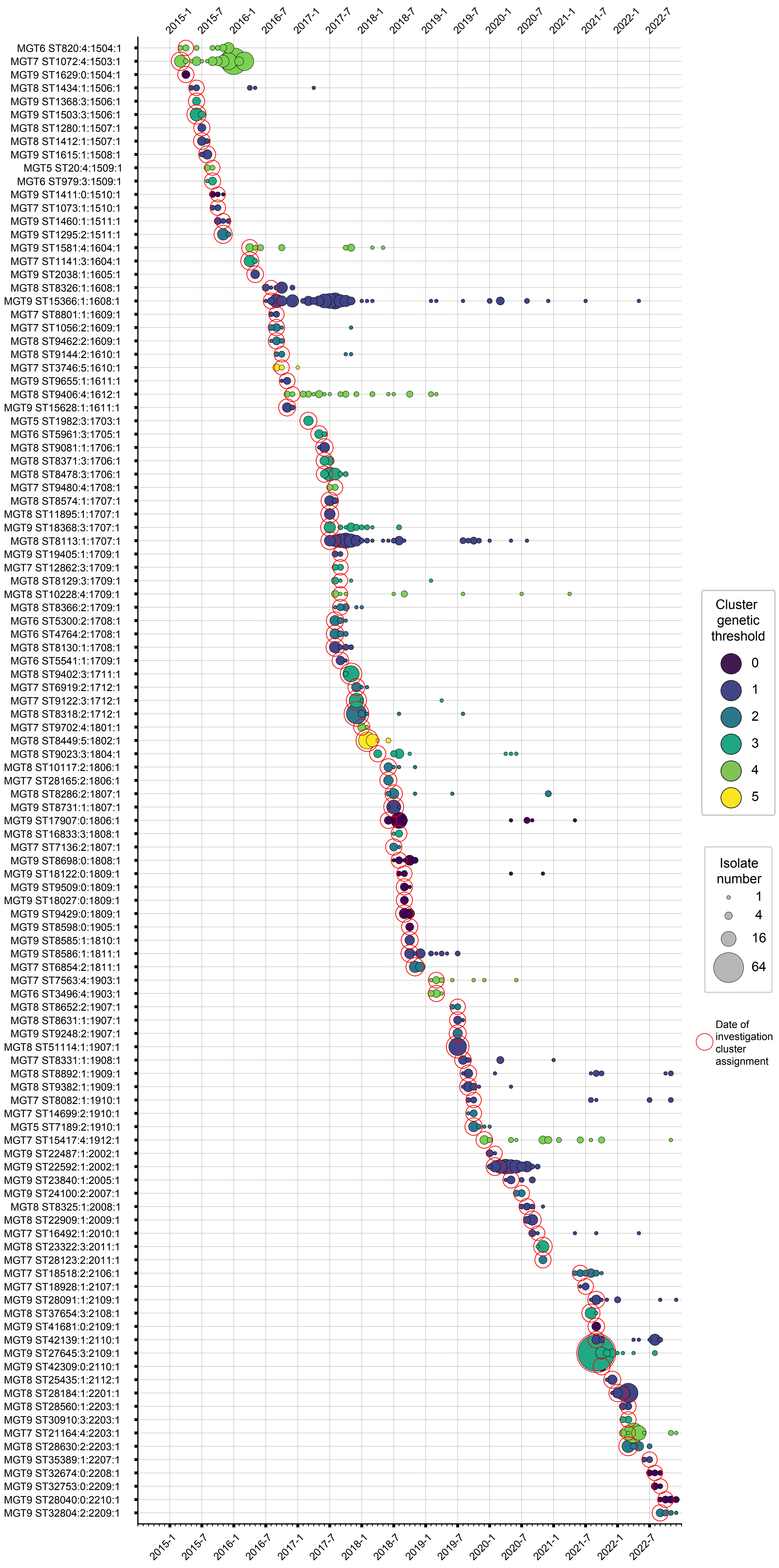

Supplementary Figure 1. Investigation clusters identified from the UK *Salmonell enterica* serovar Typhimurium dataset over 8 years. X axis is date of collection by month. Y axis is investigation cluster with MGT based ID. The area of circles is proportional to number of isolates in that investigation cluster in that month. Colour represents the genetic threshold used for that investigation cluster. Red outline indicates the week in which the cluster was identified as an investigation cluster by the DODGE algorithm. All isolates from 2014 were used as the background dataset.

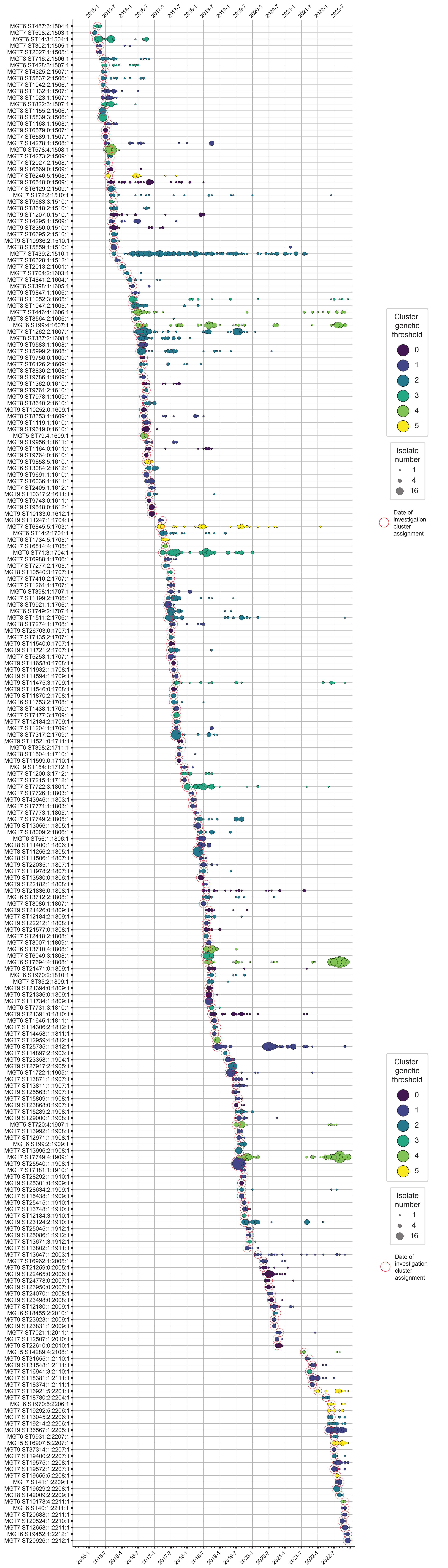

Supplementary Figure 2. Investigation clusters identified from the UK *Salmonell enterica* serovar Enteritidis dataset over 8 years. X axis is date of collection by month. Y axis is investigation cluster with MGT based ID. The area of circles is proportional to number of isolates in that investigation cluster in that month. Colour represents the genetic threshold used for that investigation cluster. Red outline indicates the week in which the cluster was identified as an investigation cluster by the DODGE algorithm. All isolates from 2014 were used as the background dataset.

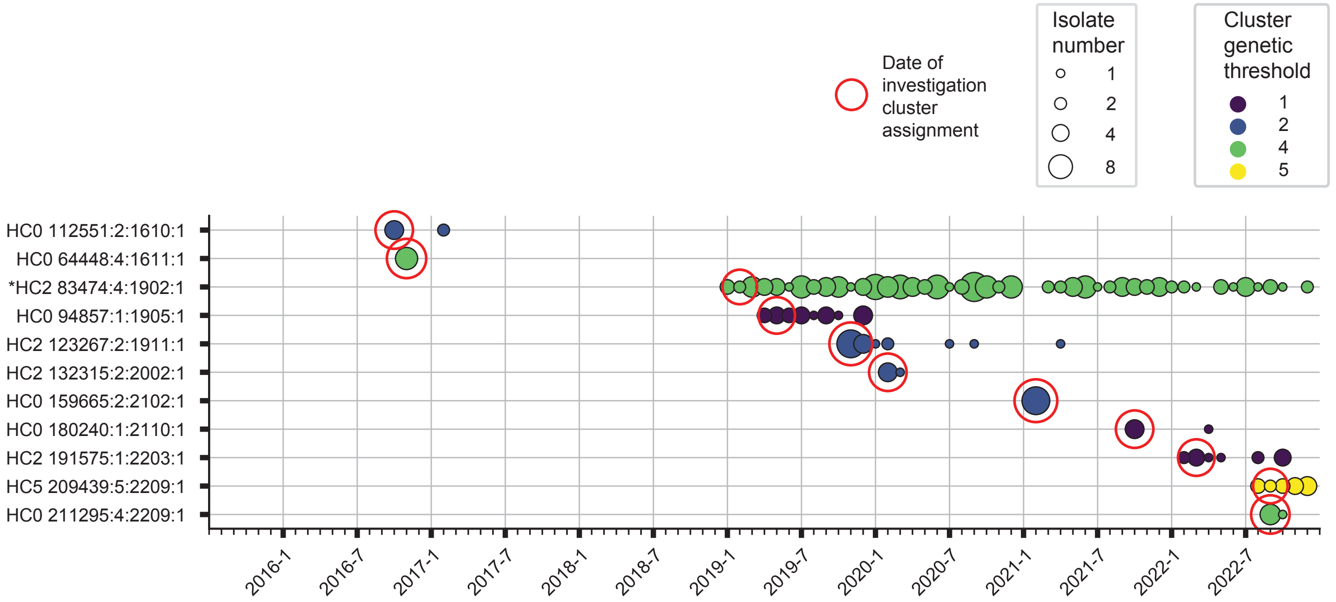

Supplementary Figure 3. Investigation clusters identified from the UK *Shigella flexneri* dataset over 7 years. X axis is date of collection by month. Y axis is investigation cluster with hierCC based ID. The area of circles is proportional to number of isolates in that investigation cluster in that month. Colour represents the genetic threshold used for that investigation cluster. Red outline indicates the week in which the cluster was identified as an investigation cluster by the DODGE algorithm. All isolates from 2015 were used as the background dataset.

*The investigation cluster HC2 83474:4:1902:1 matches the novel MSM associated clade described in Dallman et al 2021.
